# Health belief model-based determinants of women’s participation in cervical cancer screening: a case-control study from Southeastern Turkey

**DOI:** 10.1186/s12905-026-04582-0

**Published:** 2026-05-30

**Authors:** Ufuk Acar, Feyyaz Barlas, Burcu Beyazgul, Ibrahim Koruk

**Affiliations:** 1 Departmant of Public Health, Harran University Faculty of Medicine, Central District, Şanlıurfa-Mardin Road, Haliliye, Sanliurfa 63000 Turkey; 2 Sanliurfa Provincial Health Directorate, Sanliurfa, Turkey

**Keywords:** Cervical screening, Screening uptake, Papanicolaou (Pap) test, Health belief model, Women’s health

## Abstract

**Background:**

In line with the World Health Organization’s elimination targets, HPV‑DNA-based cervical cancer screening is the standard; however, screening uptake remains suboptimal in many settings. To assess, in a centre implementing Türkiye’s national cancer screening standards, the relationship between women’s screening status and a Health Belief Model (HBM)-based, 19‑item attitude scale; and to provide clear, practice‑oriented findings on screening behaviour.

**Methods:**

In a case-control design, women aged 30-65 years were studied (November-December 2024). A total of 210 participants (1:1 screened vs. not screened) were included. The HBM‑based scale and sociodemographic variables were administered. Chi‑square tests were used for categorical variables and Mann-Whitney U tests for continuous variables. Scale scores were dichotomised at the median (low/high), and unadjusted odds ratios (ORs) were calculated from 2 × 2 tables; multivariable logistic regression was additionally used to estimate adjusted odds ratios (aORs) controlling for age, education, marital status, employment, and perceived income (two-sided *p* < 0.05).

**Results:**

Scale scores were significantly higher among women who had undergone screening [median (IQR)]: perceived severity 22(4) (*p* < 0.001, *r* = 0.339), perceived susceptibility 12(7) (*p* < 0.001, *r* = 0.314), perceived barriers/self-efficacy 17(3) (*p* < 0.001, *r* = 0.355), perceived benefits 19(4) (*p* = 0.03, *r* = 0.176), and total score 71(9) (*p* < 0.001, *r* = 0.483). Women with low scores had higher odds of not being screened: severity OR = 3.04, susceptibility OR = 3.33, barriers/self‑efficacy OR = 2.86, total score OR = 4.41 (all *p* < 0.001); benefits OR = 1.17 (not significant, *p* = 0.68). Associations remained significant after adjustment for sociodemographic characteristics: low total HBM score aOR = 4.50 (2.46-8.26, *p* < 0.001); low perceived severity aOR = 3.73, low perceived susceptibility aOR = 4.92, low perceived barriers/self-efficacy aOR = 3.10 (all *p* < 0.01).

**Conclusion:**

The HBM‑based attitude scale indicates that perceived threat (severity, susceptibility) and barriers/self‑efficacy are strongly associated with women’s screening behaviour; the total belief score discriminates better than individual subscales. Findings support culturally sensitive communication and service arrangements focused on reducing barriers and enhancing self‑efficacy to improve screening participation.

**Supplementary Information:**

The online version contains supplementary material available at 10.1186/s12905-026-04582-0.

## Background

Cervical cancer is the fourth most common cancer among women worldwide; in 2022 approximately 660,000 new cases and 350,000 deaths were reported (the vast majority in low‑ and middle‑income countries) [1]. This burden reflects marked global inequities in access to vaccination, screening and effective treatment. In response, the World Health Organization (WHO) has adopted the elimination targets 90-70-90: 90% of girls fully vaccinated against HPV by age 15; 70% of women screened with a high‑performance test at ages 35 and 45; and 90% of women with pre‑cancer or cancer receiving appropriate treatment [2, 3].

WHO’s 2021 guideline recommends HPV‑DNA testing as the preferred screening method over visual inspection with acetic acid (VIA) or cytology (Pap smear); 5-10‑year intervals are advised for HPV‑negative women, with more frequent intervals for women living with HIV [4]. Türkiye’s national cancer screening programme recommends HPV‑DNA testing every 5 years for women aged 30-65, with appropriate triage (cytology/colposcopy) for positive results [5]. At the country level, approximately 2,532 new cervical cancer cases and 1,245 deaths are estimated annually, underscoring the need to achieve elimination targets [6].

Within the Eastern Mediterranean Region (EMR), cervical cancer ranks sixth among women; in 2022 there were about 16,000 new cases and > 10,000 deaths. Substantial gaps remain in scaling up screening and vaccination across countries in the region. Participation in screening is shaped by individual‑ and system‑level factors including sociocultural norms, privacy/embarrassment, fear of pain, structural barriers to access and health literacy [7].

The Health Belief Model (HBM) is frequently used to explain the psychosocial determinants of screening behaviour. Multiple syntheses indicate that perceived benefits and perceived barriers are among the strongest correlates of behaviour, with perceived susceptibility and perceived severity also playing important roles [8, 9]. Testing this theoretical framework-aligned with an HPV‑DNA‑based national screening strategy-within a neighbouring sociocultural context can inform the design of behavioural interventions and the integration of primary and secondary prevention [2–5, 7].

This study aims to examine, among women aged 30–65 years, the association between having undergone cervical cancer screening (within a national programme prioritising HPV‑DNA with cytology triage where appropriate) and HBM components-perceived susceptibility, perceived severity, perceived benefits and perceived barriers/self‑efficacy; to assess discriminative performance; and to identify which perceptual domains should be prioritised to enhance screening participation. Using a case-control design, we seek to add practice‑oriented findings with high policy transferability to the limited research from our region.

## Methods

### Study design and reporting

This research is an observational epidemiological case-control study. Reporting follows the STROBE checklist for case-control studies [10].

In this setting, a case-control design was selected to efficiently examine psychosocial determinants of cervical cancer screening uptake within a limited field period. Participation in screening represents a behavioural outcome shaped by individual perceptions and contextual factors rather than a clinical endpoint. Recruiting women who had and had not undergone screening from the same KETEM service pathway allowed a focused comparison of Health Belief Model-based perceptions while reducing heterogeneity related to access to healthcare services and information. Cases were defined as women who had not previously undergone cervical cancer screening, whereas controls were women who had undergone screening at the centre during the same period. A 1:1 case-control ratio was used to enhance comparability and statistical efficiency.

### Research area: structure of KETEM

The study was conducted at the Cancer Early Diagnosis, Screening and Education Centre (KETEM) affiliated with the Şanlıurfa Haliliye District Health Directorate. KETEMs provide community‑based cancer screening, counselling and education; they increase access to screening through annual population‑based plans and primary‑care referrals. Breast, cervical and colorectal screening are implemented according to national standards; HPV-DNA primary cervical screening algorithms and triage processes (cytology/colposcopy) are standardized in routine practice. KETEM thus enables access to women who have and have not undergone screening within the same service pathway, supporting a comparative sample and reducing misclassification through standardized workflows. In Türkiye’s national programme, women aged 30-65 years are offered HPV‑DNA testing every 5 years with triage for positives; KETEMs are the front‑line implementers of this programme [11, 12].

### Study population, sample and participant selection

The target population comprised women aged 30-65 years presenting to KETEM. Between November and December 2024, consecutive attendees who met eligibility criteria were invited. The case group included women who had not previously undergone cervical cancer screening; the control group comprised women who had a cervical smear at the centre. While Türkiye’s national cervical cancer screening programme is HPV-DNA-based with cytology/colposcopy triage, the operational indicator of screening behaviour during fieldwork was ‘having a smear taken’ at KETEM. This approach reflects routine service workflows, in which HPV-DNA testing and cytology sampling are delivered within the same service pathway and represent the most consistently documented, participant-recognisable indicator of screening uptake in centre records. As clarified above, this operational definition does not represent a deviation from national HPV-DNA-based guidelines but rather captures their routine implementation at the service level, where HPV-DNA testing and cytology sampling are operationally integrated within KETEM workflows. A 1:1 sampling ratio was achieved, yielding 210 participants (105 cases, 105 controls) for analysis.

Eligibility criteria;

- *Inclusion*: age 30-65 years; provision of informed consent; ability to understand and respond in Turkish.
- *Exclusion*: prior hysterectomy or a diagnosis of cervical cancer; cognitive/communication limitations precluding questionnaire completion; repeat attendance during the study period (duplicate record).

### Sample size and power

Following a pilot, G*Power 3.1 was used to estimate the required sample size for two independent groups assuming a small effect size (0.20), α = 0.05 and power = 0.95; a minimum of 105 participants per group was targeted and achieved [13].

### Variables and measurements

Data were collected using a study-specific questionnaire comprising two components: (i) a section on sociodemographic and sexual-reproductive health characteristics, with items compiled by the authors for the present study and informed by the relevant literature; and (ii) the previously published Health Belief Model (HBM)-based Sexually Transmitted Infections Attitude Scale [14]. The full English-language version of the questionnaire is provided as Supplementary File 1.

- *Dependent variable*: “Having undergone cervical cancer screening”, operationalized as having a cervical smear at KETEM (yes/no).
- Although HPV-DNA testing constitutes the primary screening modality in the national programme, Pap smear sampling remains a routine and participant-salient component of screening delivery and was therefore used as the operational definition of screening uptake in this study.
- *Independent variables*: total and subscale scores of the Health Belief Model (HBM)-based Sexually Transmitted Infections Attitude Scale (19 items; perceived susceptibility, perceived severity, perceived benefits, perceived barriers/self‑efficacy) and sociodemographic/sexual‑reproductive characteristics (age, education, marital status, perceived income, sexual activity, family‑planning method use). On the barriers/self‑efficacy subscale, higher scores indicate fewer barriers/greater self‑efficacy. The scale was developed in Turkish; the original study reported Cronbach’s α=0.74; internal consistency (subscales and total) was re‑evaluated in the present study [14]. In the present sample, internal consistency was acceptable. Cronbach’s α coefficients were 0.71 for perceived severity, 0.76 for perceived susceptibility, 0.68 for perceived barriers/self-efficacy, 0.69 for perceived benefits, and 0.73 for the total score.

### Data collection

Trained researchers administered a face‑to‑face questionnaire at KETEM (approximately 10-12 min). Screening status was cross‑checked with centre records wherever feasible and duplicates were prevented. Data confidentiality was ensured using anonymized identifiers.

### Ethical approval and administrative permissions

Ethical approval was obtained from the Harran University Faculty of Medicine Clinical Research Ethics Committee (session 02.12.2024, decision HRU/24.19.34). Institutional permission was granted by the Sanlıurfa Provincial Health Directorate (16.10.2024, decision 380284). Written informed consent was obtained from all participants. All procedures complied with the Declaration of Helsinki and institutional standards.

### Statistical analysis

Analyses were performed using IBM SPSS Statistics, Version 26.0 (IBM Corp., Armonk, NY, USA). Normality of continuous variables was assessed using the Kolmogorov-Smirnov test, distributional plots, and skewness/kurtosis. Categorical variables were summarized as n (%); continuous variables as mean ± SD and median (IQR) as appropriate. Chi‑square tests were used for categorical comparisons and Mann-Whitney U tests for continuous variables (effect sizes were reported as rank-biserial correlation (r) for Mann-Whitney U test). To examine associations between scale scores and screening status, scores were dichotomized at the median (low/high) and odds ratios (ORs) were calculated. Receiver Operating Characteristic (ROC) analysis was used to evaluate the discriminative performance of the total score (area under the curve, AUC) and to identify an optimal cut‑off.

To address potential confounding by sociodemographic characteristics, multivariable binary logistic regression was additionally performed with not having undergone cervical screening as the outcome. Two complementary models were fitted: Model 1 entered the dichotomised total HBM score; Model 2 entered the four dichotomised subscale scores jointly. Both models were adjusted for age (continuous), education (three categories: no or limited schooling, primary or secondary, high school or above), marital status (married vs. unmarried), employment status, and perceived income (three categories: low, moderate, high). Education and perceived income were collapsed to ensure adequate cell counts and to avoid sparse-data bias. Adjusted odds ratios (aORs) with 95% confidence intervals are reported. Model performance was assessed by the likelihood-ratio χ², Nagelkerke R², and the Hosmer-Lemeshow goodness-of-fit test. Statistical significance was set at two‑sided *p* < 0.05.

## Results

A total of 210 women were included; 105 had undergone cervical cancer screening and 105 had not. The median age was 45.0 years (30.0-63.0) among those who had been screened and 42.0 years (30.0-65.0) among those not screened, with no difference by age (*p* = 0.21). Groups were also similar by education (*p* = 0.26), gainful employment (*p* = 0.64), marital status (*p* = 0.07) and perceived income (*p* = 0.30) (Table 1).

**Table 1:** Sociodemographic characteristics of participants by cervical cancer screening status

Variable	Screened *n* (%)	Not screened *n* (%)	χ²	*p*
Education level				
No formal education	28 (58.3)	20 (41.7)	6.46	0.26
Literate, no schooling	7 (30.4)	16 (69.6)		
Primary education	32 (52.5)	29 (47.5)		
Secondary education	18 (56.3)	14 (43.7)		
High school	12 (46.2)	14 (53.8)		
University or higher	8 (40.0)	12 (60.0)		
**Employment**				
Employed	9 (42.9)	12 (57.1)	0.21	0.64
Unemployed	96 (50.8)	93 (49.2)		
**Marital status**				
Single/Divorced/Separated/Widowed	6 (28.6)	15 (71.4)	3.39	0.07
Married	99 (52.4)	90 (47.6)		
**Perceived income**				
High	33 (57.9)	24 (42.1)	2.73	0.30
Moderate	42 (49.4)	43 (50.6)		
Low	30 (44.1)	38 (55.9)		

Screened = had undergone cervical cancer screening; Not screened = had not undergone cervical cancer screening. Values are presented as n (%). Chi-square test was used

Screening uptake did not differ by sexual activity (active: 50.0% vs. inactive: 50.0%; *p* = 1.00) or by use of any family‑planning method (yes: 50.0% vs. no: 50.0%; *p* = 1.00). There was a difference across method types (*p* = 0.04), driven by the condom and tubal ligation groups (Table 2).

**Table 2:** Sexual activity and family-planning use in relation to cervical screening status

Variable	Screened *n* (%)	Not screened *n* (%)	χ²	*p*
Sexual activity				
Active	79 (50.0)	79 (50.0)	0.00	1.00
Inactive	26 (50.0)	26 (50.0)		
**Use of any family-planning method**				
Yes	43 (50.0)	43 (50.0)	0.00	1.00
No	62 (50.0)	62 (50.0)		
**Type of family-planning method** ^**†**^				
Oral contraceptive pill	6 (75.0)	2 (25.0)	10.10	0.04
Condom	6 (28.6)	15 (71.4)		
Intrauterine device	17 (45.9)	20 (54.1)		
Withdrawal	6 (60.0)	4 (40.0)		
Tubal ligation	8 (80.0)	2 (20.0)		

† Fisher’s exact test recommended due to small expected counts; the overall difference was mainly contributed by the condom and tubal-ligation groups. Screened = had undergone cervical cancer screening; Not screened = had not undergone cervical cancer screening

Scores on the HBM‑based Sexually Transmitted Infections Attitude Scale (subscales and total) were significantly higher in women who had undergone screening than in those who had not [median (IQR); mean ± SD]: For perceived severity, 22 (4); 21.8 ± 2.7 vs. 20 (3); 20.2 ± 2.8 among those not screened (*p* < 0.001, *r* = 0.339). For perceived susceptibility, 12 (7); 12.2 ± 4.4 vs. 10 (5); 9.8 ± 4.0 (*p* < 0.001, *r* = 0.314); for barriers/self-efficacy, 17 (3); 16.8 ± 2.7 vs. 16 (3); 15.3 ± 2.6 (*p* < 0.001, *r* = 0.355); for benefits, 19 (4); 19.1 ± 3.3 vs. 19 (4); 17.8 ± 3.3 (*p* = 0.03, *r* = 0.176). The total score was 71 (9); 69.9 ± 7.8 among screened vs. 64 (11); 63.0 ± 7.9 among non-screened (*p* < 0.001, *r* = 0.483) (Table 3).

**Table 3:** Attitude scale scores by cervical screening status

Subscale	Screened median (IQR)	Not screened median (IQR)	U statistic	*p*	rank-biserial *r*
Perceived severity	22 (4)	20 (3)	3646.00	< 0.001	0.339
Perceived susceptibility	12 (7)	10 (5)	3781.00	< 0.001	0.314
Perceived barriers/self-efficacy^†^	17 (3)	16 (3)	3555.00	< 0.001	0.355
Perceived benefits	19 (4)	19 (4)	4541.00	0.03	0.176
Total score	71 (9)	64 (11)	2848.00	< 0.001	0.483

Values are median (IQR). Mann-Whitney U test was applied. Rank-biserial correlation (r) is reported as an effect size (small ≈ 0.1, medium ≈ 0.3, large ≥ 0.5). † For the barriers/self-efficacy subscale, higher scores indicate fewer barriers and greater self-efficacy

Odds of not being screened (median‑based low vs. high) were higher for women with low scores on the subscales and the total score: perceived severity OR = 3.04 (95% CI 1.72-5.38), perceived susceptibility OR = 3.33 (1.88-5.92), barriers/self‑efficacy OR = 2.86 (1.61-5.05) and total score OR = 4.41 (2.46-7.87). For benefits, OR = 1.17 (0.68-2.00) and the association was not significant (*p* = 0.68) (Table 4).

**Table 4:** Unadjusted odds of not being screened for cervical cancer by low vs. high attitude scale scores (median split)

Subscale	Not screened *n*/*N* (%)-Low	Not screened *n*/*N* (%)-High	OR (low vs. high)	95% CI	*p*
Perceived severity	73/118 (61.9)	32/92 (34.8)	3.04	1.72–5.38	< 0.001
Perceived susceptibility	75/120 (62.5)	30/90 (33.3)	3.33	1.88–5.92	< 0.001
Perceived barriers/self-efficacy^†^	75/124 (60.5)	30/86 (34.9)	2.86	1.61–5.05	< 0.001
Perceived benefits	58/112 (51.8)	47/98 (48.0)	1.17	0.68-2.00	0.68
Total score	75/113 (66.4)	30/97 (30.9)	4.41	2.46–7.87	< 0.001

† On this subscale, higher scores indicate fewer barriers and greater self‑efficacy

Event = not screened; Reference = high score (≥ median); Low score = < median. ORs and 95% CIs are from 2 × 2 tables

Findings were robust to adjustment for sociodemographic characteristics in multivariable logistic regression. In Model 1, women with a low total HBM score retained markedly higher odds of not having undergone screening compared with those scoring above the median (aOR = 4.50, 95% CI 2.46-8.26, *p* < 0.001), independent of age, education, marital status, employment status, and perceived income. In Model 2, in which the four HBM subscales were entered jointly, low perceived severity (aOR = 3.73, 95% CI 1.74-8.00, *p* < 0.001), low perceived susceptibility (aOR = 4.92, 95% CI 2.43-9.94, *p* < 0.001), and low perceived barriers/self-efficacy (aOR = 3.10, 95% CI 1.44-6.66, *p* = 0.004) remained independently associated with not having been screened, whereas the perceived benefits subscale was not (aOR = 0.73, 95% CI 0.37-1.45, *p* = 0.370). Among sociodemographic covariates, marital status was independently associated with screening uptake in Model 1 (married vs. unmarried: aOR = 0.28, 95% CI 0.09-0.88, *p* = 0.029) but did not retain statistical significance in Model 2; this attenuation is descriptive only and should not be interpreted as evidence of mediation given the cross-sectional case–control design. Both models showed acceptable global fit (Hosmer–Lemeshow *p* = 0.21 and 0.16, respectively) and meaningful discrimination (Nagelkerke R² = 0.215 and 0.320). The crude and adjusted odds-ratio estimates were of similar magnitude and direction, indicating that the associations between HBM components and cervical cancer screening uptake were not materially overestimated by uncontrolled sociodemographic confounding (Table 5).

**Table 5:** Multivariable logistic regression of factors associated with not having undergone cervical cancer screening, adjusted for sociodemographic characteristics

Variable	Adjusted OR	95% CI	*p*
Model 1: Total HBM score			
Low total HBM score (ref = high)	4.50	2.46-8.26	< 0.001
Age (per 1 year)	0.97	0.92-1.01	0.107
Education: No or limited schooling (ref = high school or above)	0.84	0.31-2.27	0.726
Education: Primary or secondary (ref = high school or above)	0.69	0.28-1.69	0.415
Married (ref = unmarried)	0.28	0.09-0.88	0.029
Employed (ref = unemployed)	1.27	0.41-3.93	0.673
Perceived income: Moderate (ref = high)	1.40	0.64-3.06	0.397
Perceived income: Low (ref = high)	1.72	0.78-3.79	0.180
**Model 2: HBM subscales (entered jointly)**			
Low perceived severity (ref = high)	3.73	1.74-8.00	< 0.001
Low perceived susceptibility (ref = high)	4.92	2.43-9.94	< 0.001
Low perceived barriers/self-efficacy† (ref = high)	3.10	1.44-6.66	0.004
Low perceived benefits (ref = high)	0.73	0.37-1.45	0.370
Age (per 1 year)	0.98	0.93-1.02	0.342
Education: No or limited schooling (ref = high school or above)	0.37	0.13-1.09	0.070
Education: Primary or secondary (ref = high school or above)	0.50	0.20-1.26	0.141
Married (ref = unmarried)	0.49	0.15-1.56	0.225
Employed (ref = unemployed)	1.31	0.41-4.18	0.647
Perceived income: Moderate (ref = high)	1.55	0.68-3.55	0.301
Perceived income: Low (ref = high)	2.15	0.91-5.06	0.080

Outcome: not having undergone cervical cancer screening (event = not screened). Education was collapsed into three categories owing to small expected counts; the highest-attainment category served as the reference. Perceived income was collapsed into three ordered levels (high/moderate/low) for the same reason. Marital status was dichotomised as married vs unmarried (single, divorced, separated or widowed), consistent with Table 1. † On the perceived barriers/self-efficacy subscale, higher scores indicate fewer barriers and greater self-efficacy. Model 1: likelihood-ratio χ²(8) = 36.84, *p* < 0.001; Nagelkerke R² = 0.215; Hosmer-Lemeshow *p* = 0.21. Model 2: likelihood-ratio χ²(11) = 57.63, *p* < 0.001; Nagelkerke R² = 0.320; Hosmer-Lemeshow *p* = 0.16

**Fig. 1:**
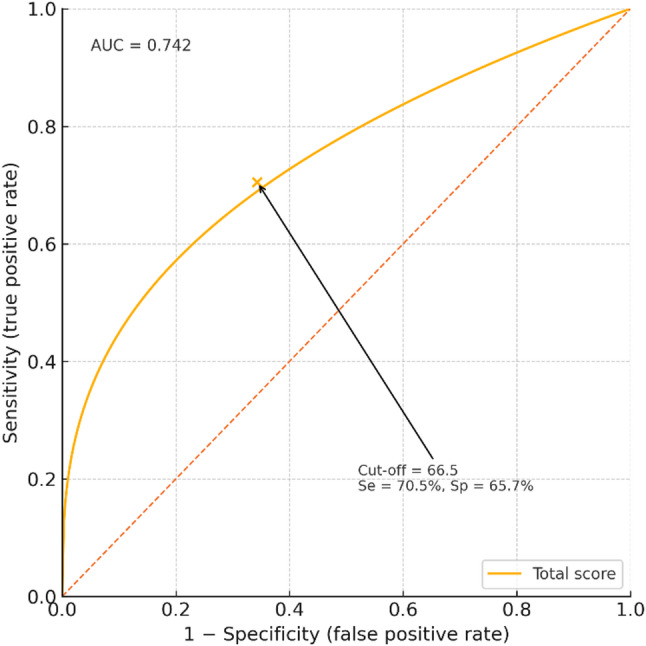
Receiver operating characteristic (ROC) curve of the HBM‑based attitude total score for discriminating women who had undergone cervical cancer screening (AUC = 0.742). At the 66.5 cut‑off, sensitivity = 70.5% and specificity = 65.7%; the dashed diagonal denotes the no‑discrimination line

Using the total score, ROC analysis showed AUC = 0.742 (*p* < 0.001). An optimal cut‑off of 66.5 yielded specificity 65.7% and sensitivity 70.5% for predicting having undergone screening (Fig. 1).

## Discussion

This study demonstrates that Health Belief Model-based perceptions particularly perceived threat (severity and susceptibility) and barriers/self-efficacy are strongly associated with women’s participation in cervical cancer screening within a national programme context. The findings indicate a clear and consistent association between HBM components and screening behaviour: higher perceived severity and higher perceived susceptibility, as well as lower perceived barriers/higher self‑efficacy, were each associated with a significant reduction in the odds of not undergoing a screening test. The total scale score provided the strongest discrimination (OR = 4.41). An AUC of 0.742 for the total score suggests that concise, scale-based assessments may support prioritisation within service workflows, although they should not replace clinical decision-making. Importantly, these associations persisted after multivariable adjustment for age, education, marital status, employment, and perceived income, with adjusted odds ratios that were similar in magnitude and direction to the crude estimates. This indicates that the relationship between HBM perceptions and cervical cancer screening uptake in this setting is not materially explained by the measured sociodemographic characteristics, and supports interpreting these perceptions as plausible behavioural targets in their own right. Overall, the pattern aligns with WHO’s elimination strategy (90-70-90) and Türkiye’s HPV‑DNA primary screening approach, offering behavioural targets to improve participation across the Eastern Mediterranean Region (EMR) [2–5, 7].

Perceived threat, encompassing both severity and susceptibility, emerged as a key determinant of screening participation in this study [8, 9]. Regional evidence points in the same direction: in Iran, a study of women’s genital warts prevention behaviours identified perceived severity, susceptibility and self‑efficacy as direct determinants [15]. Among women living with HIV in rural Uganda, participation in cervical cancer screening-conducted in line with national guidance using VIA and/or cytology-was independently predicted by perceived susceptibility and perceived severity [16]. Taken together, these data support targeting counselling and reminder messages-within KETEM services-in ways that ethically and accurately reinforce perceived threat, thereby facilitating behaviour change.

Barriers and self-efficacy emerged as central, modifiable determinants of screening behaviour with direct implications for service delivery. Self‑efficacy is theoretically a core driver of behaviour change [17] and is positioned as a key leverage point in contemporary HBM formulations [18]. In the EMR, salient barriers include privacy, fear of pain/embarrassment, logistics of scheduling/transport, and access to female providers; among young adults in Gulf countries, awareness of screening and vaccination remains limited and perceived barriers are substantial [7, 19]. Our results therefore highlight the potential impact of service arrangements such as female provider options, flexible appointments, on‑site screening teams, and counselling/reminders to reduce barriers and enhance self‑efficacy. Although perceived benefits followed the expected direction in our data, the association did not reach statistical significance; this may reflect masking by barriers/self‑efficacy or a ceiling effect in this sample. Even so, the literature consistently supports systematic effects of benefits and barriers on preventive behaviours [8, 9]. Notably, the perceived benefits subscale also failed to reach significance in the multivariable model (aOR = 0.73, *p* = 0.370), in contrast with the three other HBM domains, which retained robust independent associations. The convergence of the unadjusted and adjusted analyses on this single null finding strongly supports a ceiling-effect interpretation: with median scores at or near the upper end of the scale and limited dispersion in this population, the perceived benefits dimension appears to lack sufficient variability to discriminate screening behaviour, rather than being unimportant in principle. This pattern reinforces the conclusion that, in settings where awareness of the value of screening is already broadly endorsed, programmatic gains are most likely to come from reducing tangible barriers and strengthening self-efficacy, not from additional benefits-focused messaging.

From a programme standpoint, HPV‑DNA primary screening remains the foundation of Türkiye’s standards; while VIA/cytology are still used in resource‑constrained settings, WHO’s 2021 guideline recommends HPV‑DNA testing as the preferred method [4, 5, 11]. In the present study, screening behaviour was operationalised as having a Pap smear at KETEM; interpretation should therefore consider the primacy of HPV-DNA testing and subsequent triage steps within Türkiye’s national cervical cancer screening algorithm [4, 5, 11]. Given marked EMR‑wide variation in cervical cancer burden and participation [7], our findings have practical value for region‑tailored communication materials and access‑facilitating service designs.

Regarding ROC performance, a 66.5‑point threshold provided 70.5% sensitivity and 65.7% specificity; such a criterion can be used for prioritisation, but only alongside field protocols that account for the costs of misclassification [20]. Embedding brief, scale‑based assessments into routine counselling could standardise risk communication and contribute to programme goals [2, 3, 5, 7].

### Strengths and limitations

Conducting the study within KETEM, the frontline programme implementer, enabled concurrent access to women who had and had not undergone a screening test through the same entry point, likely reducing misclassification. The sample size was pre‑planned and achieved, and the HBM‑based attitudes were measured multidimensionally, with additional ROC assessment of discrimination.

By design, a case-control study yields odds ratios (ORs); relative risks cannot be directly estimated. Dichotomising scale scores at the median can entail information loss and may inflate apparent effect sizes. In addition, the case-control design does not allow a clear assessment of the temporal sequence between health beliefs and cervical cancer screening behaviour. Health Belief Model-based perceptions were measured at a single time point and may have been influenced by prior interactions with healthcare services, including previous screening experiences or counselling encounters. This raises the possibility of reverse causality, whereby participation in screening may shape certain beliefs rather than beliefs exclusively determining screening behaviour.

The single‑centre setting and reliance on self‑report introduce potential biases, and some potential confounders (e.g. HPV vaccination status, prior abnormal Pap results) may not have been fully accounted for. Accordingly, the findings should be interpreted as evidence of association rather than causation, and their applicability beyond similar programme-based settings should be considered with caution. The analytical strategy combined median-based 2 × 2 odds ratios - chosen to prioritise interpretability and direct service-level applicability - with multivariable logistic regression adjusted for the principal sociodemographic characteristics. Median-based categorisation, however, can entail information loss and modest residual misclassification at the cut-point. Sample size constraints precluded inclusion of a wider set of potential confounders or interaction terms (e.g., HPV vaccination history, prior abnormal cytology, parity, distance to KETEM); residual confounding by these unmeasured factors cannot be excluded. These limitations warrant cautious generalisation.

### Conclusions and recommendations

Conclusions. Perceived threat (severity, susceptibility) and barriers/self‑efficacy are key determinants of women’s participation in cervical cancer screening; the total belief score discriminates screening behaviour better than individual subscales (AUC = 0.742; OR = 4.41). This pattern provides a strong basis for behaviourally targeted strategies to increase participation across the EMR [2, 3, 5, 7, 11]. Along with this, the following recommendations can be listed in line with the aims and objectives of the study:

Service-level interventions:

- Integrate barrier-reduction and self-efficacy-enhancing measures into routine services (privacy-protecting processes, access to female providers, rapid scheduling, on-site screening teams).

Communication and counselling strategies:

- Deploy evidence-based, culturally attuned messages that ethically strengthen perceived threat.
- Emphasise self-efficacy through structured counselling and reminder systems.

Programme-level planning:

- Pilot the use of the total scale score for prioritisation within defined protocols.
- Integrate brief scale-based assessments into KETEM workflows.
- Encourage multicentre studies to enhance generalisability.
- Future research may employ multicentre designs with multivariable analyses and incorporate variables such as HPV vaccination and history of abnormal Pap results.

## Supplementary Information

Supplementary Material 1.

Supplementary Material 2

## Data Availability

The data that support the findings of this study are available from the corresponding author, [UA], upon reasonable request.

## Abbreviations

EMR: Eastern Mediterranean Region
HBM: Health Belief Model
HPV: Human papillomavirus
HPV-DNA: Human papillomavirus deoxyribonucleic acid
HSGM: General Directorate of Public Health
KETEM: Cancer Early Diagnosis, Screening and Education Centre
Pap: Papanicolaou
VIA: Visual inspection with acetic acid
WHO: World Health Organization
