# Supplementary material for: Health belief model-based determinants of women’s participation in cervical cancer screening: a case-control study from Southeastern Turkey
Source: BMC Womens Health. 2026 May 30;26:385. doi: 10.1186/s12905-026-04582-0 (PMC13430880; doi:10.1186/s12905-026-04582-0)
Supplement: Supplementary file 1 — Supplementary Material 1. [file 12905_2026_4582_MOESM1_ESM.pdf]

Supplementary File 1

Study Questionnaire (English-language Version)

Health Belief Model-based determinants of women's participation in cervical cancer screening: A case-control study from southeastern Turkey

Questionnaire No.: ________________ Neighbourhood: ________________

Part I. Sociodemographic and Sexual-Reproductive Health Characteristics

No.	Question	Response options
**1.**	How old are you (completed years)?	________________ years
**2.**	What is your level of education?	1. Illiterate 2. Literate (no formal schooling) 3. Primary school graduate 4. Secondary (middle) school graduate 5. High school graduate 6. University or higher
**3.**	Are you currently engaged in any income-generating work?	1. Yes (______________________________) 2. No
**4.**	What is your marital status?	1. Never married 2. Married 3. Divorced / separated 4. Widowed
**5.**	Do you have any children?	1. Yes 2. No
**6.**	In your opinion, is your total household income sufficient to meet your needs?	1. Very sufficient 2. Sufficient 3. Moderate 4. Insufficient 5. Very insufficient
**7.**	Are you sexually active?	1. Yes 2. No
**8.**	Do you use any family-planning method?	1. Yes - if yes, which method (________________) 2. No
**9.**	Have you had a cervical (Pap) smear taken?	1. Yes 2. No

Part II. Health Belief Model-based Sexually Transmitted Infections Attitude Scale

Source: Beyazgül B, Koruk F, Koruk İ. Development of a scale for attitude toward sexually transmitted infections based on the Health Belief Model. Eur J Obstet Gynecol Reprod Biol. 2024;298:42-48. doi:10.1016/j.ejogrb.2024.04.036.

Response format (5-point Likert): 1 = Strongly disagree | 2 = Disagree | 3 = Partially agree | 4 = Agree | 5 = Strongly agree

On the perceived barriers/self-efficacy subscale, higher scores indicate fewer perceived barriers and greater self-efficacy.

No.	Statement	Strongly disagree	Disagree	Partially agree	Agree	Strongly agree
1	To prevent sexually transmitted infections, attention should be paid to contact with blood and genital discharge.					
2	To prevent sexually transmitted infections, contact with sex workers should be avoided.					
3	To prevent sexually transmitted infections, condoms should be used.					
4	If there is a sore, redness or discharge in the genital area, sexual intercourse should be avoided.					
5	To prevent sexually transmitted infections, monogamy should be preferred.					
6	I think my likelihood of contracting a sexually transmitted infection is high.					
7	I think the likelihood of having a sore or redness in my genital area within the next 5 years is high.					
8	I think the likelihood of having a discharge in my genital area is high.					
9	I think my likelihood of having a sexually transmitted infection is higher than that of other men/women.					
10	I feel that at some point in my life I will have a sexually transmitted infection.					
11	If I had a sexually transmitted infection, I would know where to go for help.					
12	If I had a sexually transmitted infection, I could go for an examination on my own.					
13	I can recognise abnormal changes that occur in my genital area.					

No.	Statement	Strongly disagree	Disagree	Partially agree	Agree	Strongly agree
**14**	Having a sexually transmitted infection is not something to be ashamed of.					
**15**	If my sexually transmitted infection is treated, my risk of infertility decreases.					
**16**	If my sexually transmitted infection is treated, my risk of death decreases.					
**17**	If my sexually transmitted infection is treated, the risk of cervical cancer for me/my partner decreases.					
**18**	If my sexually transmitted infection is treated, my chance of having children increases.					
**19**	If my sexually transmitted infection is treated, the pleasure I get from sexual intercourse increases.					

Subscale Composition

Subscale	Item numbers
**Perceived severity**	Items 1-5 (preventive attitudes)
**Perceived susceptibility**	Items 6-10
**Perceived barriers / self-efficacy**	Items 11-14 (higher scores = fewer barriers, greater self-efficacy)
**Perceived benefits**	Items 15-19

Note. The original Turkish version of the questionnaire was administered to participants. This English translation has been prepared by the authors for the purpose of supplementary documentation and reporting; it has not been formally back-translated or psychometrically re-validated, and is intended to convey the content and structure of the instrument used in the present study.
